# Supplementary material for: DNA Methylation Signatures of Breastfeeding in Buccal Cells Collected in Mid-Childhood
Source: Nutrients. 2019 Nov 17;11(11):2804. doi: 10.3390/nu11112804 (PMC6893543; doi:10.3390/nu11112804)
Supplement: Supplementary file 1 [file nutrients-11-02804-s001.zip › SupplementalFigures.pdf]

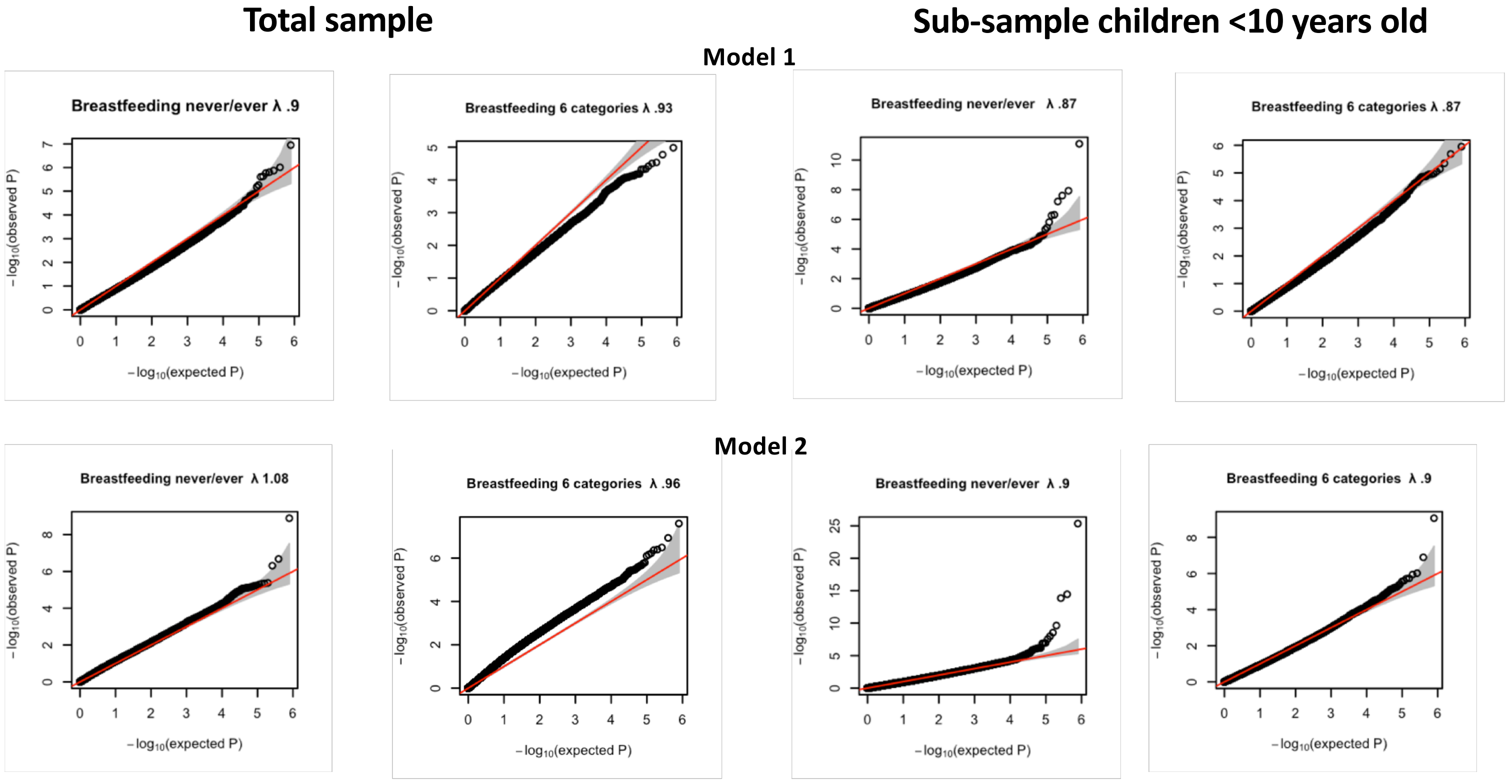

Supplemental Figure S1. QQ plots that shows epigenome-wide association study results of breastfeeding 'never'/'ever' and breastfeeding duration in 6 categories (not-breastfed; <2 weeks; 2-6 weeks; 6 weeks – 3 months; > 6 months) in discovery total sample (N=1,006) and in sub-sample of children before 10 years (N = 517). Model adjusted by sex, age, epithelium cells and NK counts and technical covariates (Model 1), adjusted for SES, maternal pre-pregnancy BMI, maternal age at birth, maternal prenatal smoking, and gestational age (Model 2).

Breastfeeding "never" / "ever"

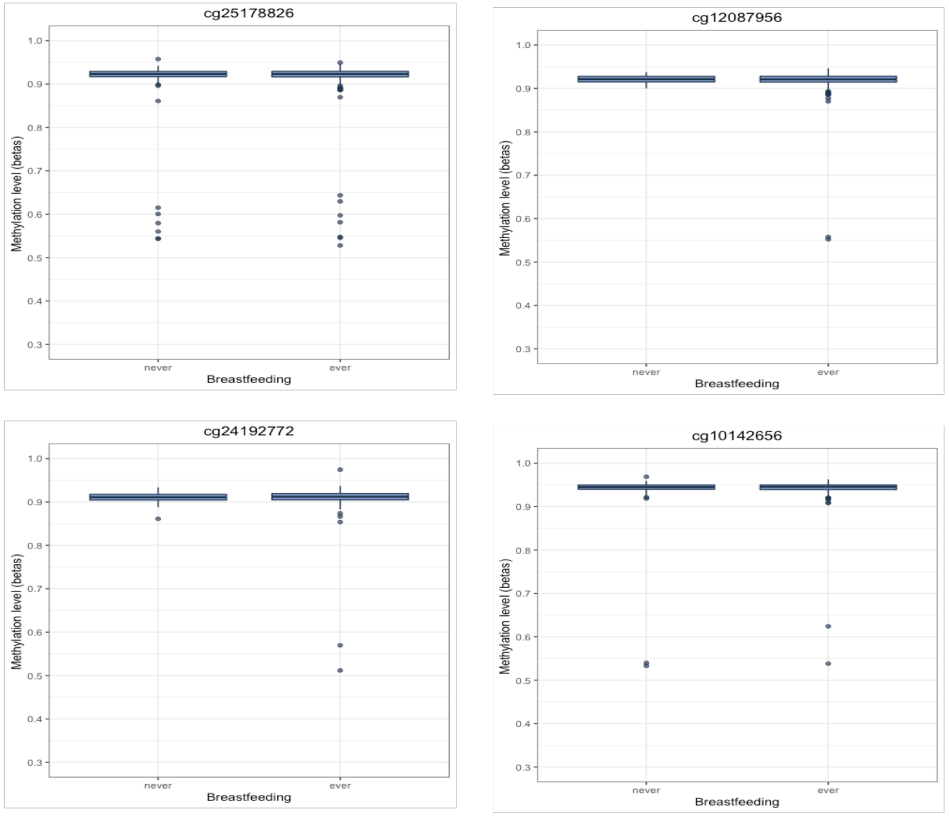

Breastfeeding 6 categories

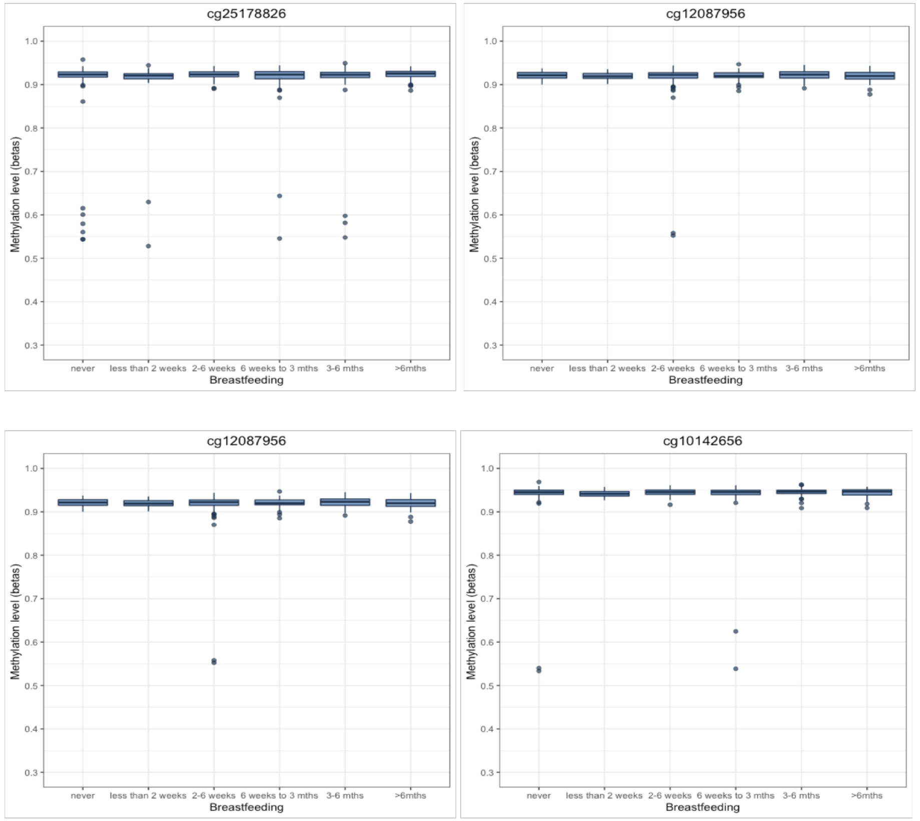

Supplemental Figure S2. Methylation level (beta) at significant CpGs in Model 1 in sub-sample of children <10 years (N = 517)

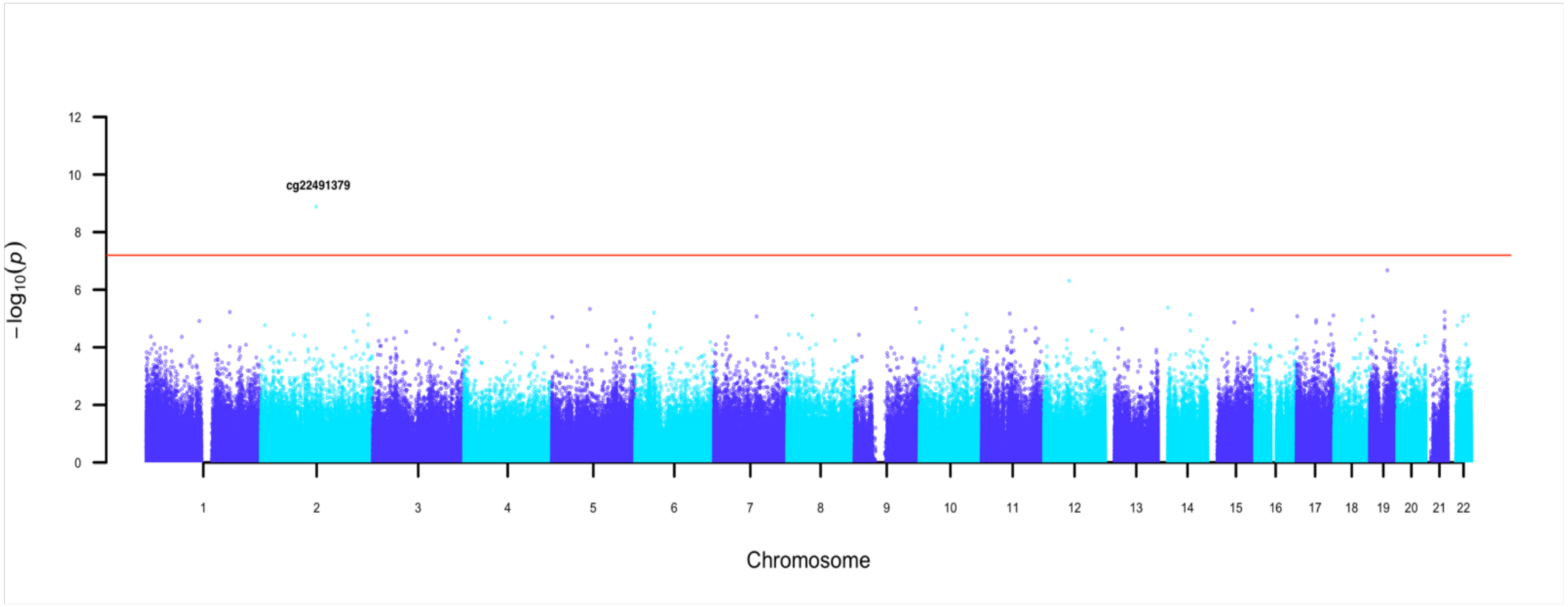

Supplemental Figure S3. Manhattan plot show the epigenome-wide association study results of breastfeeding 'never'/'ever' in discovery sample (n = 1,006). Breastfeeding as exposure is adjusted by sex, age, SES, maternal age at delivery, maternal pre-pregnancy BMI, maternal prenatal smoking, gestational age, and cell composition (adjusted model 2). In the Manhattan plot, the red line represents Bonferroni threshold (6.34e-08). Given CpG name indicates loci after outliers removal

Breastfeeding ”never” / ”ever”

Breastfeeding 6 categories

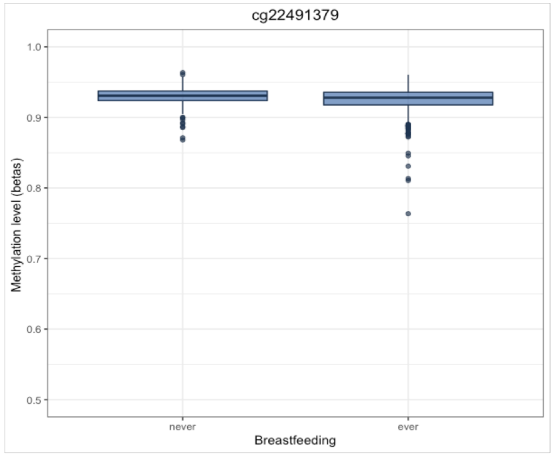

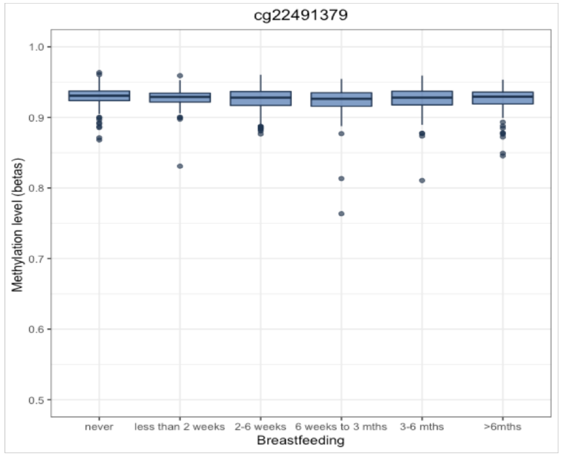

Supplemental Figure S4. Methylation level (beta) at significant CpG in Model 2 in total sample (N = 1,006)

Breastfeeding ”never” / ”ever” Breastfeeding 6 categories

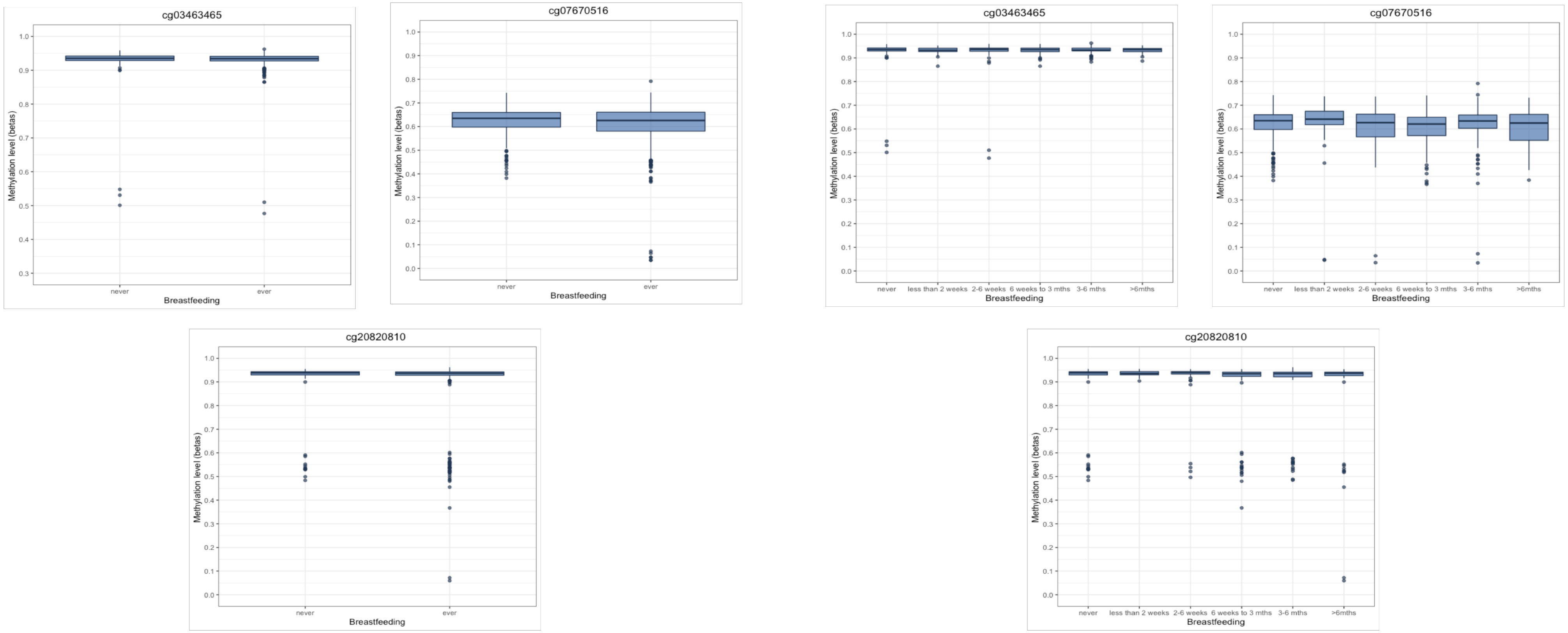

Supplemental Figure S5. Methylation level (beta) at significant CpGs in Model 2 in sub-sample of children <10 years (N = 517) with outliers excluded from further replication

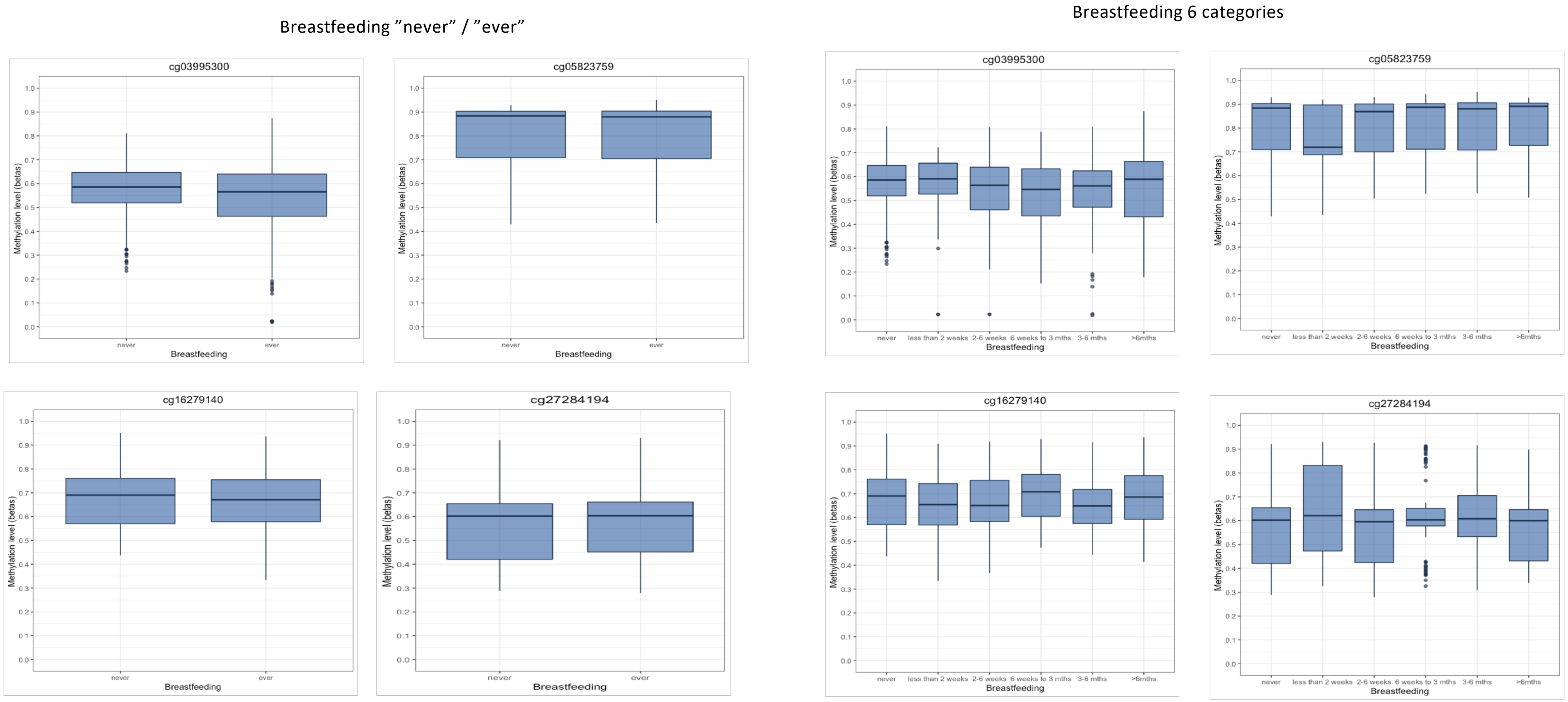

Supplemental Figure S6. Methylation level (beta) at significant CpGs in Model 2 in sub-sample of children <10 years included in replication study

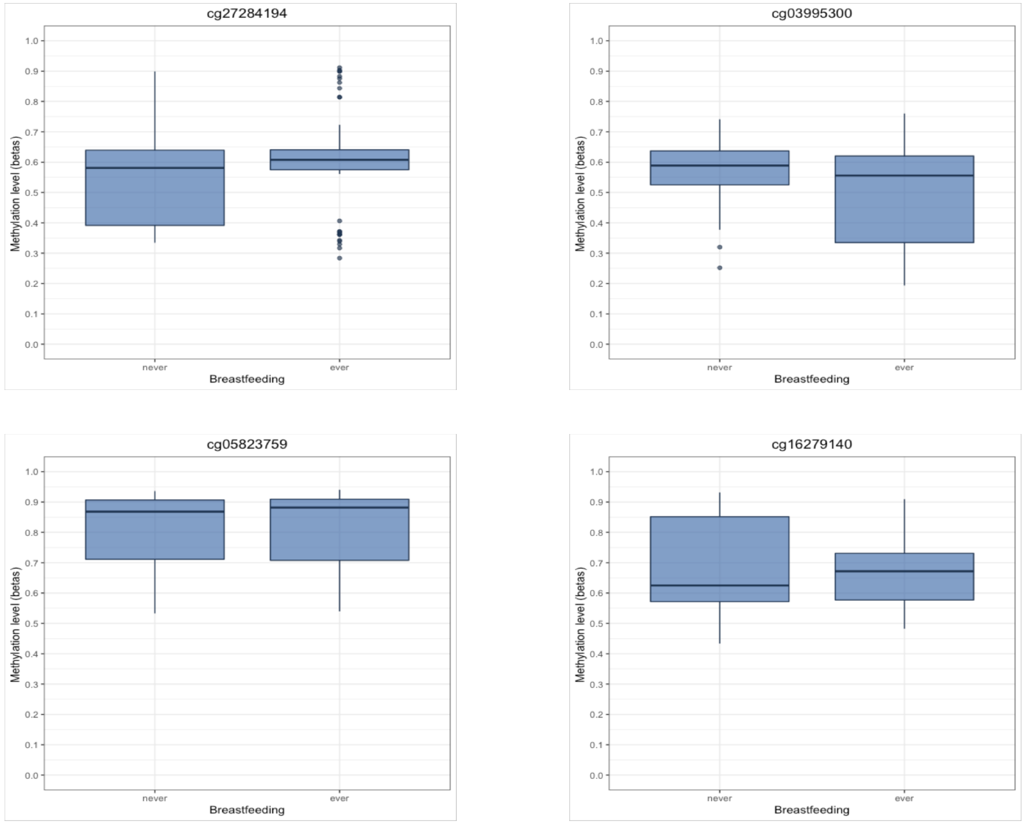

Supplemental Figure S7. Methylation level (beta) at significant CpGs in Model 2 in NTR replication study (N =100)
