# Supplementary material for: DNA Methylation Signatures of Breastfeeding in Buccal Cells Collected in Mid-Childhood
Source: Nutrients. 2019 Nov 17;11(11):2804. doi: 10.3390/nu11112804 (PMC6893543; doi:10.3390/nu11112804)
Supplement: Supplementary file 1 [file nutrients-11-02804-s001.zip › SupplementalTablesS1_7.docx]

**DNA methylation signatures of breastfeeding in buccal cells collected in mid-childhood**

*Supplementary materials*

**Supplemental Table S1.** Overview of previous EWASs of breastfeeding in humans

|  | Authors | Country | Type of study | Sample size and setting | Breastfeeding codification | DNA source | EWAS measurement | Age at DNA collection | Covariates | Main results |
| --- | --- | --- | --- | --- | --- | --- | --- | --- | --- | --- |
| 1 | Obermann-Borst et al., 2013 | Netherlands | Candidate | 120 | Ranges 0, >1 - <1, >1 - 3, >3-6 and > 6 months | peripheral blood | mass spectrometry - based quantification of PCR amplicons from bisulfate-converted DNA | mean age 1.4 years | bisulfite batch, CpG site, maternal education and smoking at birth, sex, birth weight, current BMI and serum leptin | Percentage points in methylation per increment in breastfeeding duration category: -0.6 (95% CI: -1.19; -0.01) |
| 2 | Rossnerova et al, 2013 | Czech Republic | EWAS/breastfeeding as covariate | 200 (100 asthmatic and 100 controls) | Continuous duration in months | peripheral blood | Infinium Human Methylation27 BeadChip, genome -wide DNAm | mean age 11.6 years | none | Pooling asthmatic group and controls, breastfeeding apparently associated with patterns of overall DNAm, although no statistical test was performed |
| 3 | Soto-Ramirez et al, 2013 | England | EWAS/breastfeeding as covariate | 245 females | Continuous duration in weeks | peripheral blood | Infinium Human Methylation450 BeadChip, genome-wide DNAm | 18 years | none | There was an interaction between breastfeeding and mQTLs regarding the methylation level of 10 CpG sites located at the 17q21 locus (*IL4R* gene) |
| 4 | Tao et al., 2013 | USA | candidate | 639 (all breast cancer) | 0 = Ever, 1 = Never | paraffin-embedded tumour tissue | Methylation-specific qPCR using bisulfite converted DNA, genome-wide DNAm | mean age 57.6 years | menopause status (stratification), age, education, race, and estrogen receptor status | Odds ratio of *CDKN2A (*p16*)* promoter methylation was 2.75 (95% CI: 1.14; 6.62) times higher in never breastfed women, but only in the premenopausal group |
| 5 | Sherwood et al, 2019 | UK | EWAS/effect of breastfeeding | 297 at 10 years and 305 at 18 years | Continuous duration in weeks. Categories: exclusive (until formula feed and/or solid foods were introduced) and total breastfeeding. | peripheral blood | Infinium Human Methylation450 BeadChip, genome-wide DNAm | 10 years and 18 years | child sex, birth weight, gestational age, serum leptin (ng/ml at either 10 or 18 years), BMI (at either 10 or 18 years), maternal socioeconomic status, maternal smoking at birth, parity, maternal age, cell proportions | At 10 years, the total breastfeeding duration was significantly associated with DNAm at four CpG sites in LEP. Two of the CpGs were available in 18-year samples, which showed significant differences with 10-year samples For the 18-year samples, there were no significant associations seen at any of the 16 LEP CpG sites. No significant DMPs at both age samples. Five significant DMRs were identified using comb-p. No statistically significant effects of significant CpGs as mediator between total breastfeeding duration and the early transient overweight trajectory. |
| 6 | Naumova et al, 2019 | Russia | EWAS/effect of breastfeeding | 37 | Continuous duration in weeks | peripheral blood | EPIC, genome-wide DNAm | mean age 25.7 months | age, sex, blood cell count | Methylation level of 4276 CpGs had a statistically significant positive (2201 CpGs) or negative (2075 CpGs) association with the duration of breastfeeding (partial correlation controlling for the age and the gender, and the individual variability in blood cell count). About 20% of the total variability in methylation levels might be attributed to an impact of breastfeeding and its duration. About 10% of the variability in methylation level of CpGs seems to be associated with the variability in cell-type composition. The CpG sites associated with breastfeeding were related to 2635 genes. Among the biological processes there were those related with the cellular signaling systems and function of the immune and nervous systems. The oxytocin-signaling pathway was at the top of the list of those pathways. |
| 7 | Hartwig et al 2019 | UK | EWAS/effect of breastfeeding | 640 at 7 years, 709 at 15-17 years  ALSPAC | Different categorizations:  0 = Ever, 1 = Never; ranges (0=never, 1= 1 day to 3 months, 2=3.01 to 6 months, 3= 6.01 to 12 months; 4=more than 12 months; duration in months | peripheral blood | Infinium Human Methylation450 BeadChip, genome-wide DNAm |  | White ethnic background, family SES, parity, height pre-pregnancy weight, age at birth, folic acid supplementation, maternal smoking during pregnancy, type of delivery, gestational age, birthweight, sex, age at blood collection, cell counts, surrogate variables | Methylation level of cg11414913, located on chromosome 1, was associated with breastfeeding exposure in group of 7 years old children. |

**Supplemental Table S2.** EWAS Model Equations

The two main models were fitted for breastfeeding ‘never’/‘ever’ and breastfeeding duration (6 categories):

| Model and parameters | Equation | Discovery study | Replication study |
| --- | --- | --- | --- |
| **Model 1 (basic)**  CpGi = DNA methylation 𝛽-value at methylation site i, 𝛼 = the intercept, *Breastfeedingever* is coded as 0=never, and 1=ever; *Breastfeedingduration* is coded as 1=never, 2=<0.5 months, 3=0.5-1.5 months; 4 = 1.5-3 months; 5 = 3-6 months; 6 = >6 months; sex is coded 0 for males and 1 for females, age = the age at DNA methylation in years, Epi = count of epithelial cells, NK = count of natural killer cells, arrow row = the row of the sample on the Illumina EPIC Beadchip (ranging from 1 to 8), plate 1…plate13 = bisulfite plates, and 𝜀 is residual. | 1) CpGi = 𝛼 + 𝛽*breastfeedingever*𝑥 *Breastfeedingever* + 𝛽sex 𝑥 *sex* + 𝛽age 𝑥 *age* + 𝛽*Epi* 𝑥 *Epi*+ 𝛽*NK* 𝑥 *NK*  + 𝛽*array row* 𝑥 *array row* + 𝛽*sampleplate1* 𝑥 *plate1*… + ….𝛽*sampleplate13* 𝑥 *plate13* + 𝜀​ | Total  Sub-sample <10 years  Sub-sample >10 years |  |
|  | 2) CpGi = 𝛼 + 𝛽*breastfeedingduration*𝑥 *Breastfeedingduration* + 𝛽sex 𝑥 *sex* + 𝛽age 𝑥 *age* + 𝛽*Epi* 𝑥 *Epi*+ 𝛽*NK* 𝑥 *NK*  + 𝛽*array row* 𝑥 *array row* + 𝛽*sampleplate1* 𝑥 *plate1*… + ….𝛽*sampleplate13* 𝑥 *plate13 +* 𝜀​ | Total  Sub-sample <10 years |  |
| **Model 2 (adjusted)**  CpGi = DNA methylation value at methylation sites of selected CpGs, 𝛼 = the intercept, *Breastfeedingever* is coded as 0=never, 1=ever; *Breastfeeding_6categories* is coded as 1=never, 2=<0.5 months, 3=0.5-1.5 months; 4 = 1.5-3 months; 5 = 3-6 months; 6 = >6 months; sex is coded 0 for males and 1 for females, age is the age at DNA methylation in years, SES is parental SES, matsmok=maternal prenatal smoking, matBMI=maternal pre-pregnancy BMI, agemot1=maternal age at delivery, GA=gestational age, Epi=estimated proportion of epithelial cells, NK is estimated proportion of natural killer cells, array row is the row of the sample on the Illumina EPIC Beadchip (ranging from 1 to 8), plate 1…plate13 are bisulfite plates, and 𝜀 is residual. | 1) CpGi = 𝛼 + 𝛽*breastfeedingever*𝑥 *Breastfeedingever* + 𝛽sex 𝑥 *sex* + 𝛽age 𝑥 *age* + 𝛽SES𝑥 *SES*+ 𝛽matsmok𝑥 *matsmok*+ 𝛽agemot1 𝑥 *agemot1*+ 𝛽matBMI 𝑥 *matBMI+* 𝛽GA𝑥 *GA* +𝛽 *Epi* 𝑥 *Epi*+ 𝛽*NK* 𝑥 *NK*  + 𝛽*array row* 𝑥 *array row* + 𝛽*sampleplate1* 𝑥 *plate1*… + ….𝛽*sampleplate13* 𝑥 *plate13* + 𝜀​ | Total  Sub-sample <10 years  Sub-sample >10 years | NTR  (for significant in discovery study CpGs)  ALSPAC  (for significant in discovery study CpGs) |
|  | 2) CpGi = 𝛼 + 𝛽*breastfeedingduration*𝑥 *Breastfeeding_6categories* + 𝛽sex 𝑥 *sex* + 𝛽age 𝑥 *age* + 𝛽SES𝑥 *SES*+ 𝛽matsmok𝑥 *matsmok*+ 𝛽agemot1 𝑥 *agemot1*+ 𝛽matBMI 𝑥 *matBMI+* 𝛽GA𝑥 *GA* +𝛽 *Epi* 𝑥 *Epi*+ 𝛽*NK* 𝑥 *NK*  + 𝛽*array row* 𝑥 *array row* + 𝛽*sampleplate1* 𝑥 *plate1*… + ….𝛽*sampleplate13* 𝑥 *plate13* + 𝜀​ | Total  Sub-sample <10 years |  |

**Supplemental Table S3.** Associations of covariates with breastfeeding duration (six categories)

|  | Breastfeeding 6 categories (N=823) | | | | | |
| --- | --- | --- | --- | --- | --- | --- |
|  | B | Std. Error | 95% Wald Confidence Interval | |  |  |
|  |  |  | Lower | Upper | df | Sig. |
| Child's age at methylation | 0.091 | 0.0452 | 0.003 | 0.180 | 1 | 0.044 |
| Sex (0 males, 1 females) | 0.194 | 0.1678 | -0.135 | 0.523 | 1 | 0.247 |
| **Parental SES** | **0.353** | 0.0998 | 0.158 | 0.549 | 1 | 0.0004 |
| **Maternal prenatal smoking (0/1)** | **-0.733** | 0.3160 | -1.353 | -0.114 | 1 | 0.020 |
| **Gestational age (z-scores)** | **-0.194** | 0.0904 | -0.372 | -0.017 | 1 | 0.031 |
| Maternal age at birth (z-scores) | -0.016 | 0.0922 | -0.197 | 0.164 | 1 | 0.860 |
| Maternal pre-pregnancy BMI (z-scores) | -0.117 | 0.0753 | -0.265 | 0.030 | 1 | 0.119 |
| Count of epithelium cells | 1.271 | 1.1115 | -0.908 | 3.449 | 1 | 0.253 |
| Count of natural killer cells | 14.779 | 9.8198 | -4.467 | 34.026 | 1 | 0.132 |

To test associations of breastfeeding with covariates to be included in the EWAS GEE model the following model was applied: Breastfeeding 6 categories = 𝛼 + 𝛽sex 𝑥 *sex* + 𝛽age 𝑥 *age* + 𝛽SES𝑥 *SES*+ 𝛽matsmok𝑥 *matsmok*+ 𝛽agemot 𝑥 *agemot1*+ 𝛽matBMI 𝑥 *matBMI+* 𝛽GA𝑥 *GA +* 𝛽 *Epi* 𝑥 *Epi*+ 𝛽*NK* 𝑥 *NK*  + + 𝜀​, where SES = parental SES; matsmok = maternal prenatal smoking (0/1); agemot = maternal age at delivery (z-scores); matBMI = maternal pre-pregnancy BMI; GA = gestational age (z-scores); age = child’s age at DNA methylation; sex = child’s sex (0=males, 1=females); Epi =count of epithelial cells; NK = count of natural killers.

**Supplemental Table S4.** *Discovery study subsample of children <10 years population characteristics*

|  | **Breastfeeding** | **Breastfeeding** |  |
| --- | --- | --- | --- |
|  | **Never** | **Ever** | **P-value** |
| n | 141 | 378 |  |
| Age at methylation (mean (sd)) | 7.8 (1.14) | 7.9 (1.13) | 0.341 |
| Sex (n(%) males | 75 (53.2%) | 181 (48.1%) | 0.306 |
| Zygosity (n(%) monozygotic) | 125 (90%) | 325 (86.4%) | 0.267 |
| Parental SES |  |  |  |
| *lower secondary educational level* | 15 (12.2%) | 18 (5.7%) | <0.001 |
| *upper secondary education level* | 57 (43.5%) | 89 (28.2%) |  |
| *higher vocational level* | 45 (34.4%) | 118 (37.3%) |  |
| *scientific level* | 13 (9.9%) | 91 (28.8%) |  |
| Gestational age (mean(sd)) | *36.4 (1.89)* | *35.5 (2.67)* | 0.000 |
| Maternal age at birth (mean(sd)) | 32.1 (4.48) | 30.9 (4.2) | 0.005 |
| Maternal BMI before pregnancy (mean(sd)) | *23.7 (3.97)* | *24.06 (3.99)* | 0.412 |
| Maternal smoking during pregnancy (n(%)) | 26 (20.2%) | 32 (8.9%) | 0.001 |
| Birthweight (mean(sd)) | *2450.3 (446.83)* | *2324.4 (552.2)* | 0.020 |
| Breastfeeding duration |  |  |  |
| *no*​ | 141 | 0 |  |
| *less than 2 weeks*​ |  | 39 (10.4%) |  |
| *2 to 6 weeks*​ |  | 104 (27.7%) |  |
| *6 weeks to 3 months*​ |  | 86 (22.9%) |  |
| *3 to 6 months*​ |  | 84 (22.3%) |  |
| *more than 6 months*​ |  | 63 (12.2%) |  |
| Epithelium cells count (mean (sd)) | .7921 (.142) | .7946 (.120) | 0.841 |
| Natural killer cells count (mean (sd)) | .0308 (.0136) | .0313 (.0123) | 0.703 |

**Supplemental Table S5.** *Discovery study subsample of children >10 years population characteristics*

|  | **Breastfeeding** | **Breastfeeding** |  |
| --- | --- | --- | --- |
|  | **Never** | **Ever** | **P-value** |
| n | 124 | 365 |  |
| Age at methylation (mean (sd)) | 11.06 (.73) | 11.2 (.72) | .088 |
| Sex (n(%) males | 63 (50.8%) | 168 (46%) | .825 |
| Zygosity (n(%) monozygotic) | 99 (79.8%) | 288 (78.9%) | .825 |
| Parental SES |  |  |  |
| *lower secondary educational level* | 14 (11.6%) | 31 (8.6%) | .101 |
| *upper secondary education level* | 42(34.7%) | 114 (31.6%) |  |
| *higher vocational level* | 44(36.4%) | 116(32.1%) |  |
| *scientific level* | 21(17.4%) | 100 (27.7%) |  |
| Gestational age (mean(sd)) | *36 (2.5)* | *36 (2.5)* | .957 |
| Maternal age at birth (mean(sd)) | 31.8(4.6) | 31.4(4.1) | .453 |
| Maternal BMI before pregnancy (mean(sd)) | *24.9(3.9)* | *24.4(4.2)* | .228 |
| Maternal smoking during pregnancy (n(%)) | 7 (6.4%) | 16 (5%) | .564 |
| Birthweight (mean(sd)) | *2419.8 (443.9)* | *2466.8 (555.4)* | .404 |
| Breastfeeding duration |  |  |  |
| *no*​ | 124 (100%) |  |  |
| *less than 2 weeks*​ |  | 36 (9.9%) |  |
| *2 to 6 weeks*​ |  | 85 (23.3%) |  |
| *6 weeks to 3 months*​ |  | 95 (26.0%) |  |
| *3 to 6 months*​ |  | 64 (17.5%) |  |
| *more than 6 months*​ |  | 85 (23.3%) |  |
| Epithelium cells count (mean (sd)) | .831(.091) | .819(.103) | .232 |
| Natural killer cells count (mean (sd)) | .028(.012) | .029(.012) | .389 |

**Supplemental Table S6.** *NTR replication sample population characteristics*

|  | **Breastfeeding** | **Breastfeeding** |  |
| --- | --- | --- | --- |
|  | **Never** | **Ever** | **p-value** |
| n | 36 | 62 |  |
| Age at methylation (mean (sd)) | 7.72 | 7.32 | 0.435 |
| Sex (n(%) males) | 14 (38.9%) | 38 (61.3%) | 0.032 |
| Zygosity (n(%) monozygotic) | 100% | 100% |  |
| Parental SES |  |  |  |
| *lower secondary educational level* | 10 (27.8%) | 14 (22.6%) | 0.116 |
| *upper secondary education level* | 19 (52.8%) | 26 (41.9%) |  |
| *higher vocational level* | 4 (11.1%) | 18 (29%) |  |
| *scientific level* | 3 (8.3%) | 4 (6.5%) |  |
| Gestational age (mean(sd)) | 36 (2.3) | 37 (1.9) | 0.023 |
| Maternal age at birth (mean(sd)) | 29.9 (3.2) | 32.1 (3.9) | 0.007 |
| Maternal pre-pregnancy BMI (mean(sd)) | 23.1 (2.5) | 23.9 (3.5) | 0.214 |
| Maternal smoking during pregnancy (n(%)) | 5 (13.9%) | 6 (9.7%) | 0.524 |
| Birthweight (mean(sd)) | 2329.6 (446.6) | 2686.67 (493.7) | 0.001 |
| Breastfeeding duration |  |  |  |
| *no*​ | 36 (100%) | 0 |  |
| *less than 2 weeks*​ |  | 7 (11.3%) |  |
| *2 to 6 weeks*​ |  | 13 (21%) |  |
| *6 weeks to 3 months*​ |  | 20 (32.3%) |  |
| *3 to 6 months*​ |  | 6 (9.7%) |  |
| *more than 6 months*​ |  | 16 (25.8%) |  |
| Epithelium cells count (mean (sd)) | 0.8073 (0.0854) | 0.7869 (0.1039) | 0.321 |
| Natural killer cells count (mean (sd)) | 0.0326 (0.0109) | 0.0345 (0.0122) | 0.442 |

**Supplemental Table** **S7***. ALSPAC replication sample population characteristics*

|  | **Breastfeeding** | |  |
| --- | --- | --- | --- |
|  | **Never** | **Ever** | **p-value** |
| n | 113 | 752 |  |
| Age (mean (sd)) | 7.46 (0.14) | 7.45 (0.14) | 0.184 |
| Sex (% male) | 54 (47.8) | 379 (50.4) | 0.677 |
| Maternal education (%) |  |  | <0.001 |
| *CSE/none* | 17 (15.0) | 46 (6.1) |  |
| *Vocational* | 15 (13.3) | 39 (5.2) |  |
| *O level* | 57 (50.4) | 239 (31.8) |  |
| *A level* | 19 (16.8) | 249 (33.1) |  |
| *Degree* | 5 (4.4) | 179 (23.8) |  |
| Maternal smoking during pregnancy (%) | 20 (17.7) | 86 (11.4) | 0.082 |
| Maternal age (mean (sd)) | 29.29 (4.60) | 30.33 (4.24) | 0.017 |
| Maternal BMI (mean (sd)) | 23.77 (4.93) | 22.65 (3.46) | 0.003 |
| Gestational age (mean (sd)) | 39.35 (1.66) | 39.58 (1.50) | 0.124 |
| Bcell (mean (sd)) | 0.14 (0.03) | 0.14 (0.03) | 0.849 |
| CD4T (mean (sd)) | 0.20 (0.05) | 0.21 (0.05) | 0.384 |
| CD8T (mean (sd)) | 0.03 (0.03) | 0.04 (0.03) | 0.604 |
| Gran (mean (sd)) | 0.45 (0.08) | 0.44 (0.08) | 0.308 |
| Mono (mean (sd)) | 0.06 (0.03) | 0.06 (0.03) | 0.328 |
| NK (mean (sd)) | 0.19 (0.04) | 0.19 (0.04) | 0.962 |
